# Patients Presenting With a Clenched Fist: A Perspective From an Electrodiagnostic Laboratory

**DOI:** 10.7759/cureus.117502

**Published:** 2026-10-05

**Authors:** Lisa B Shields, Vasudeva G Iyer, Smita Ghare, Christopher B Shields

**Affiliations:** 1 Norton Neuroscience Institute, Norton Healthcare, Louisville, USA; 2 Neurodiagnostic Center of Louisville, Louisville, USA

**Keywords:** clenched fist, dystonia, electrodiagnostic study, neurology, psychiatry

## Abstract

Background/objectives: A clenched fist is a clinical sign that may have diverse etiologies, including certain psychiatric and neurological disorders, or may occur after localized trauma. The clenched fist may be partial or complete and is usually unilateral. Our objective was to determine the etiology of a clenched fist in a cohort of patients.

Methods: This is a retrospective analysis of 17 patients who were referred to our Neurodiagnostic Center for evaluation of a clenched fist over an 18.5-year period (January 1, 2008, to June 30, 2026). All patients underwent a clinical examination followed by electrodiagnostic studies, which included nerve conduction and needle electromyography (EMG), to determine the presence of neuromuscular disorders.

Results: Of the 17 patients who presented with a clenched fist, it was unilateral in 16 (94.1%) and bilateral in 1 (5.9%). Four (23.5%) patients had an underlying neurological disorder (Lewy body dementia, corticobasal degeneration, spastic dystonic hand due to a contralateral frontal lobe lesion), and one had tendinopathy. In 13 (76.5%) patients, no specific cause could be found. Most patients in the latter category either sustained a recent injury and/or surgery in the ipsilateral upper extremity with the clenched fist and/or had an extensive psychiatric history. The clenched fist was partial in 11 (64.7%) patients and complete in 6 (35.3%).

Conclusion: It is important to be aware that a clenched fist could be a manifestation of not only psychiatric conditions but also a neurological disorder. A multidisciplinary approach involving neurologists, hand surgeons, neurosurgeons, and psychiatrists is necessary for the diagnosis and management of a clenched fist. Determining the accurate etiology of the clenched fist in a timely manner may expedite appropriate treatment.

## Introduction

A clenched fist is considered a clinical sign and refers to a tendency to keep the fingers in a position of flexion intermittently or continuously. In this study, we have included patients whose main symptom was a tendency to keep the hand in a position of a clenched fist with flexion of the digits at the interphalangeal (IP) and metacarpophalangeal (MP) joints. A clenched fist may be a manifestation of certain psychiatric and/or neurological conditions or related to local trauma [1,2]. Diagnosing the underlying cause of the clenched fist in a timely manner is valuable for planning the appropriate treatment. While clenched fist is a clinical sign, the term clenched fist syndrome (CFS) has a different connotation. In 1978, Wallace and Fitzmorris coined the term S-H-A-F-T syndrome of the upper extremity, in which a patient undergoes numerous surgeries of the upper extremity as a form of Munchausen’s syndrome [3]. S-H-A-F-T is an eponym for a sad, hostile, angry, frustrated, and tenacious person and often presents with self-inflicted trauma [4]. Patients with this syndrome may be divided into two categories: those who inflict physical harm on themselves and those who posture their upper extremities in positions that could not be explained anatomically [4]. In 1980, Simmons and Vasile coined the term “clenched fist syndrome” to denote the latter group [1].

According to the DSM-5-TR (Diagnostic and Statistical Manual of Mental Disorders, Fifth Edition, Text Revision), CFS is classified as a specific motor presentation of functional neurological symptom disorder, which is an updated term for conversion disorder [5]. In the ICD-11, CFS is classified under dissociative neurological symptom disorders. CFS is only a psychiatric syndrome and does not encompass patients having organic neurological conditions. Patients with this condition often suffer from depression, schizophrenia, or obsessive-compulsive disorders [6-8]. CFS is characterized by several classic features: (1) flexion contractures of the fingers that demonstrate normal mobility under anesthesia; (2) pain, swelling, and paradoxical stiffness of the fingers; (3) often occurring after minor trauma or surgery; (4) maceration of the palm; and (5) lack of an organic etiology [4,7,8]. In CFS, the most frequent posture is rigid flexion of the proximal interphalangeal joints of the third, fourth, and fifth digits (primarily the ulnar-sided fingers) with sparing of the thumb and index finger [8-10]. This condition may affect individuals across the age spectrum and is usually unilateral [8,10]. Early recognition of CFS is important to avoid unnecessary diagnostic procedures and inappropriate surgery.

The phenomenon of clenched fist may also develop in patients with certain neurological conditions such as spasticity resulting from various causes, including stroke, head and spinal cord trauma, dystonia, corticobasal degeneration, and Parkinson’s disease [11,12]. Fist clenching may also occur in late stages of Alzheimer’s disease, which may result from physical discomfort, stress, or muscle rigidity [13,14].

Several case reports and series highlighting CFS have been published [4,6,7,9,10,15-18], but a comprehensive analysis of the etiology of clenched fist as a clinical sign is lacking. The primary objective of this study was to evaluate patients with a clenched fist with a clinical examination followed by electrodiagnostic (EDX) studies and other relevant investigations when indicated to determine the specific cause. We discuss clinical patterns, various causes, and potential treatments of clenched fist.

## Materials and methods

Under an Institutional Review Board (IRB)-approved protocol, we performed an 18.5-year (January 1, 2008, to June 30, 2026) retrospective analysis of patients with a clenched fist who were referred to our Neurodiagnostic Center for EDX studies. Our American Association of Neuromuscular and Electrodiagnostic Medicine (AANEM)-accredited Neurodiagnostic Center assesses approximately 1000 patients annually referred for EDX studies. All patients with a clenched fist underwent clinical examination and EDX studies, which included nerve conduction studies (motor and sensory) of the median and ulnar nerves as well as needle electromyography (EMG) of the hand and forearm muscles. When neurological findings raised the possibility of a central nervous system disorder, relevant imaging studies such as a brain MRI were also done. The same electromyographer, a neurologist, board-certified in electrodiagnostic medicine and clinical neurophysiology, performed the evaluations of all patients. The EMG and nerve conduction studies used Nicolet Viking proprietary EMG software (Natus Medical Incorporated, Middleton, Wisconsin). The patients did not undergo a psychiatric assessment at our Neurodiagnostic Center. We conducted a thorough review of each patient’s electronic health record through our healthcare system’s software platform Epic (Epic Systems Corporation, Verona, Wisconsin). We recorded whether a patient had a documented psychiatric history as well as the specific mental health conditions. Following the clinical examination, EDX studies, and/or a brain MRI, it was determined whether the patient had an organic basis for a clenched fist. For the patients who did not have an organic basis for a clenched fist, we labeled these patients as "idiopathic clenched fist, presumably somatic symptom disorder," with subcategories of (1) recent injury and/or surgery in the upper extremity ipsilateral to the clenched fist and (2) extensive psychiatric history. The etiological classification was determined prospectively at the time of the EDX evaluation.

Inclusion and exclusion criteria

Patients presenting with unilateral or bilateral clenched fist who were referred to our facility for nerve conduction and needle EMG studies were included. Patients who could not give a comprehensive clinical history were excluded.

Metrics collected

The clinical metrics collected included (1) demographics, (2) laterality (left, right, or bilateral), (3) partial (involving two to four digits of the hand) versus complete (all the digits), (4) physical examination, and (5) EDX findings. Neurological examination was aimed at looking for features of underlying central nervous system disorders that may cause dystonia, such as Parkinson’s disease, Lewy body dementia, corticobasal degeneration, or spasticity (stroke, brain trauma, cerebral palsy). Motor examination focused on ruling out mimics of clenched fist, such as finger drop from posterior interosseous nerve neuropathy and clawing from ulnar and/or median nerve lesions. The EDX studies, including motor and sensory conduction of the median, ulnar, and radial nerves and needle EMG of the hand and forearm muscles, were performed using the standard protocol in our laboratory [19].

Statistical analysis

The descriptive statistics (frequencies and percentages) within this analysis were calculated utilizing Microsoft Excel for Mac, Version 16.113 (Microsoft Corporation, Redmond, Washington). 

Institutional Review Board approval

The study was conducted according to the guidelines of the Declaration of Helsinki and was approved by the Institutional Review Board of the WCG. The IRB determined that our study was exempt under 45 CFR 46.104(d4). The IRB number is 20253566. The IRB approval date was September 15, 2025. All patients in this study gave their consent to undergo “clinical photographs/video and/or use my clinical data for future comparison, teaching, research, and/or scientific publication." All patients signed this consent form prior to undergoing the original EDX evaluation.

## Results

Demographics

A total of 17 patients presented with a clenched fist (Table 1). Nine (52.9%) patients were female, and the mean age was 49 years (range: 19-94 years). The clenched fist was unilateral in 16 (94.1%) patients (left: 11; right: 5) and bilateral in 1 (5.9%). A total of 13 (76.5%) patients were right-hand-dominant. The side of the clenched fist corresponded to the dominant hand in 8 (47.1%) patients. Of the 8 patients whose side of the clenched fist corresponded to the dominant hand, 4 had a right-sided clenched fist and were right-hand-dominant, 3 had a left-sided clenched fist and were left-hand-dominant, and 1 had a bilateral clenched fist and was right-hand-dominant. 

**Table 1:** Demographics, Physical Examination, Electrodiagnostic Findings, and Etiologies of Patients With a Clenched Fist Presenting to Our Neurodiagnostic Center *Incidental EDX findings. M: male; F: female; L: left; R: right; B: bilateral; C: complete; P: partial; EDX: electrodiagnostic; CTS: carpal tunnel syndrome; CuTS: cubital tunnel syndrome; ADM: abductor digiti minimi; PTSD: post-traumatic stress disorder; CRPS: complex regional pain syndrome.

Patient #	Gender (M/F)	Age (Years)	Side of Dominant Hand (L/R)	Side of Symptoms (L/R/B)	Clenched Fist (C/P)	Physical Examination Findings in Addition to a Clenched Fist	EDX Findings	Etiology
1	F	38	R	L	C	Dystonic/athetotic movements; spasticity and weakness of distal muscles; hyperactive reflexes	Decreased recruitment of motor units in distal muscles; no denervation/reinnervation changes	Dystonia from R hemispheric lesion
2	M	28	R	L	C	Normal	No denervation/ reinnervation changes	Idiopathic clenched fist, presumably somatic symptom disorder (H/O severe anxiety, stress, tearfulness, depression, hopelessness, helplessness, anhedonia)
3	M	39	R	R	P	Weakness of intrinsic hand muscles and decreased pinprick sensation in the distal forearm, palm, and ring and small fingers	No denervation/ reinnervation changes	Idiopathic clenched fist, presumably somatic symptom disorder (H/O radius fracture and surgery)
4	M	53	R	L	P	Decreased pinprick sensation over distal portion of all digits	No denervation/ reinnervation changes	Idiopathic clenched fist, presumably somatic symptom disorder (H/O forearm fracture and surgery)
5	M	53	L	L	P	Tremulous/jerky movements of fingers; decreased pinprick sensation in all digits	L CTS; L CuTS*; no denervation/ reinnervation changes	Idiopathic clenched fist, presumably somatic symptom disorder (H/O rotator cuff repair)
6	F	68	L	L	P	Diffuse weakness and rigidity with bradykinesia; hyperactive reflexes	No denervation/ reinnervation changes	Lewy body dementia
7	M	33	R	B	P	Weakness of intrinsic hand muscles and extensors of digits, more of small finger	No denervation/ reinnervation changes	Idiopathic clenched fist
8	F	32	R	L	C	Normal	No denervation/ reinnervation changes	Idiopathic clenched fist, presumably somatic symptom disorder (H/O anxiety and depression)
9	F	78	R	L	P	Diffuse weakness of distal muscles; marked rigidity in B upper extremities, more on the left side	No denervation/ reinnervation changes	Corticobasal degeneration
10	M	39	R	R	C	Weakness of intrinsic hand muscles and decreased pinprick sensation in forearm, palm, and fingers	No denervation/ reinnervation changes	Idiopathic clenched fist, presumably somatic symptom disorder (H/O arm injury with surgeries)
11	F	19	R	R	P	Normal	No denervation/ reinnervation changes	Idiopathic clenched fist, presumably somatic symptom disorder (extensive psychiatric history)
12	M	40	R	L	C	Weakness of distal muscles; decreased pinprick sensation over radial 3 digits, L hand	No denervation/ reinnervation changes	Idiopathic clenched fist, presumably somatic symptom disorder (H/O shoulder surgery)
13	F	85	L	R	P	Normal	R CTS and R CuTS^*^; no denervation/ reinnervation changes	Idiopathic clenched fist
14	F	51	R	L	P	Normal	No denervation/ reinnervation changes	Idiopathic clenched fist
15	M	34	R	L	P	Decreased pinprick sensation over small finger and ulnar side of palm	L CuTS*; no denervation/reinnervation changes	Idiopathic clenched fist, presumably somatic symptom disorder (H/O PTSD, anxiety, schizophrenia)
16	F	56	L	L	C	Normal	No denervation/ reinnervation changes	Idiopathic clenched fist
17	F	94	R	R	P	Weakness of intrinsic hand muscles	R CuTS*; no denervation/reinnervation changes	Tendinopathy (CRPS-related dystonia)

Etiology

Four (23.5%) patients had an organic etiology for the clenched fist (Tables 1, 2). Patient #1 had a clenched fist resulting from spasticity and dystonia related to a contralateral hemispheric lesion after undergoing surgery for a hypothalamic glioma at 3 years of age. Patients #6 and #9 were diagnosed with Lewy body dementia and corticobasal degeneration, respectively. A tendinopathy (secondary to complex regional pain syndrome (CRPS)-related dystonia) was the cause of the clenched fist in patient #17. No underlying central or peripheral nervous system or muscle/tendon disorder could be documented in 13 (76.5%) patients. Of these 13 patients, 5 had sustained a recent injury and/or undergone recent surgery in the upper extremity ipsilateral to the clenched fist, 4 had an extensive psychiatric history, and 4 did not have either a recent injury/surgery or an extensive psychiatric history. 

**Table 2:** Etiologies of Patients With a Clenched Fist Presenting to Our Neurodiagnostic Center

Etiology	Number of Patients (n = 17)
Known cause	4 (23.5%)
Hypothalamic glioma causing dystonia	1
Lewy body dementia	1
Corticobasal degeneration	1
Tendinopathy (secondary to complex regional pain syndrome-related dystonia)	1
Idiopathic	13 (76.5%)
Recent injury and/or surgery in upper extremity ipsilateral to clenched fist	5
Extensive psychiatric history	4

Neurological examination

The clenched fist was partial in 11 (64.7%) patients and complete in 6 (35.3%) (Figures 1, 2A,B). The physical examination findings, in addition to the clenched fist, are presented in Table 1. 

**Figure 1:**
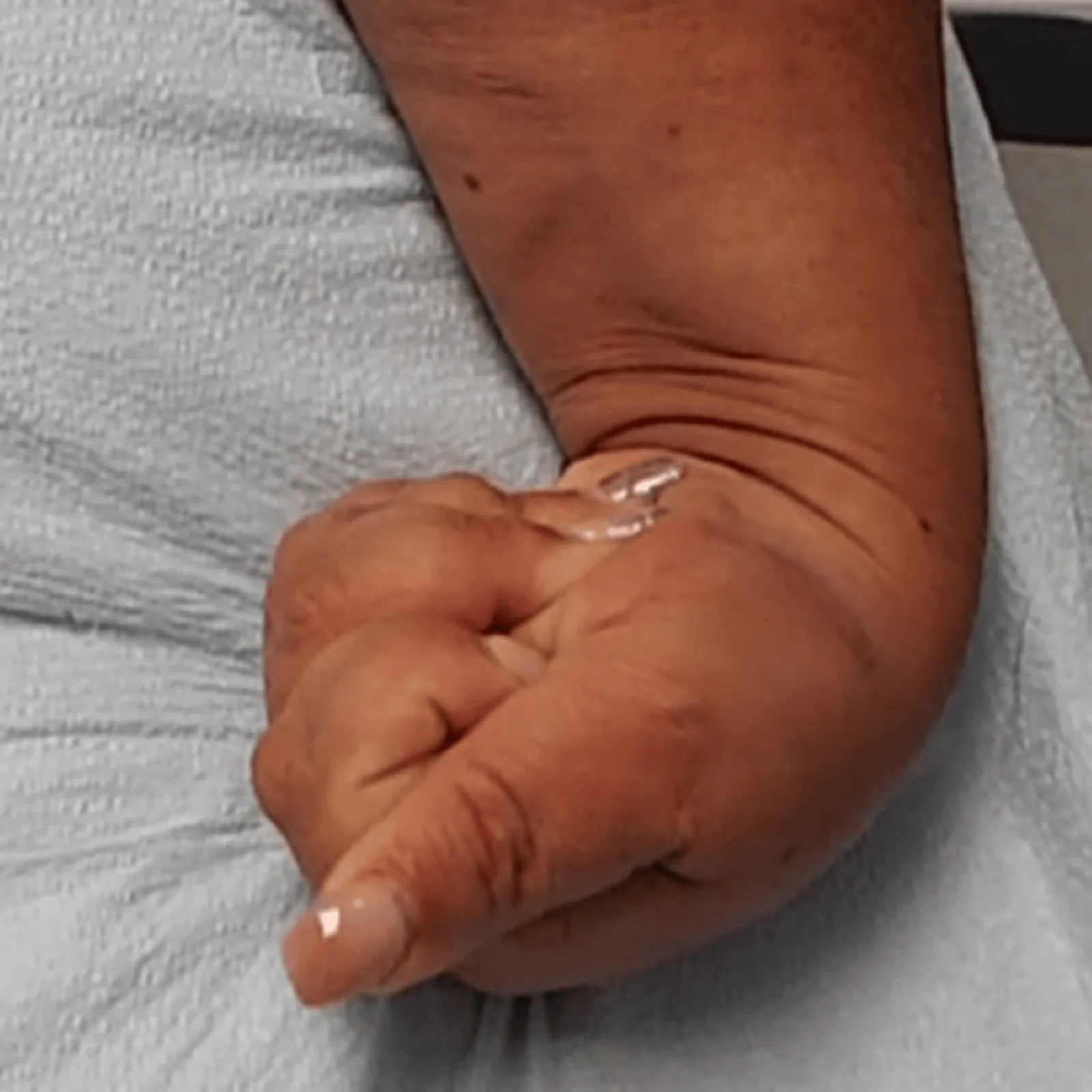
Complete Clenched Fist in Patient #1

**Figure 2:**
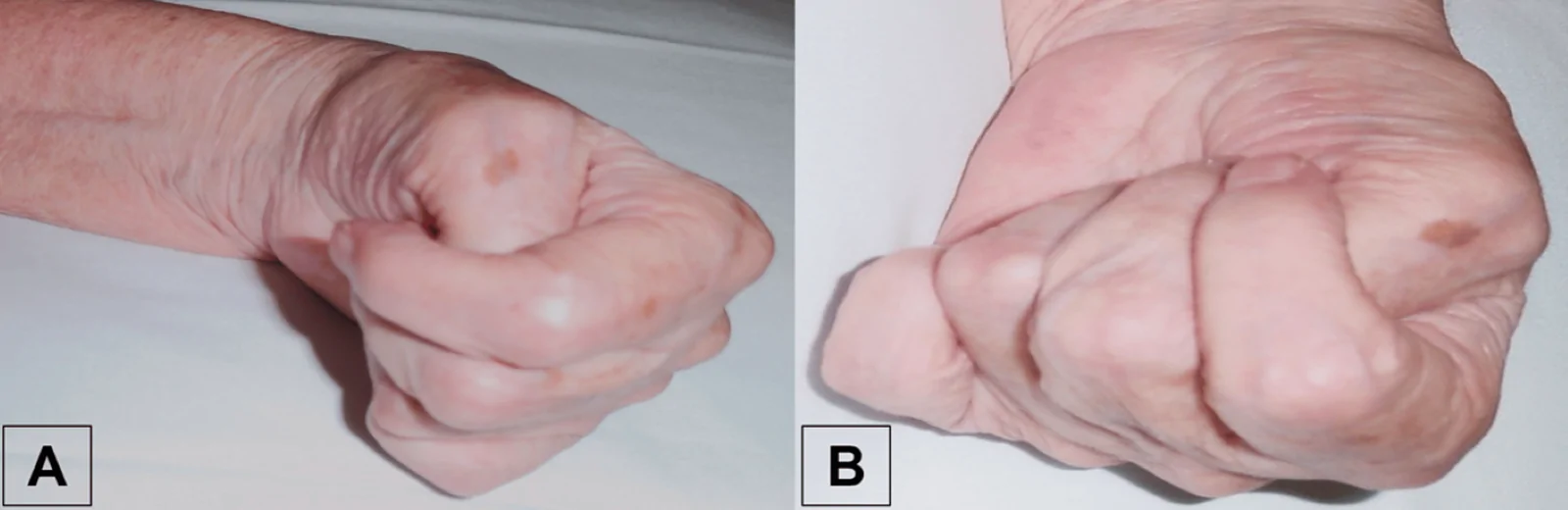
Complete Clenched Fist in Patient #2 (A, B) Different views of the complete clenched fist.

Electrodiagnostic studies

All 17 patients with a clenched fist underwent EDX studies (Table 1). Patients #5 and #13 had evidence of both moderate median nerve entrapment at the carpal tunnel and ulnar nerve entrapment at the cubital tunnel detected on EDX studies in the ipsilateral hand, and patients #15 and #17 had isolated ulnar nerve entrapment at the cubital tunnel in the ipsilateral hand; these were considered incidental findings and did not contribute to the clenched fist.

## Discussion

The differential diagnosis in patients presenting with a clenched fist includes CFS, focal dystonia seen in complex regional pain syndrome (CRPS), certain neurological disorders (cerebrovascular accident, Parkinson’s disease, and corticobasal degeneration), Dupuytren’s disease, tendonitis, rheumatoid arthritis, ulnar or median nerve compression, arthrogryposis multiplex congenita, progressive systemic sclerosis, eosinophilic fasciitis, and camptodactyly (one or more fingers (most commonly the little finger) are fixed in a bent position and cannot fully straighten) (Table 3) [4,6,10,17,20]. Local trauma may also cause a clenched fist. Injuries to tendons or their attachments, or inflammation of flexor tendon sheaths, can potentially cause flexion deformity of the fingers. Similarly, injuries that cause compartment syndrome can cause contracture of flexor muscles (Volkmann’s ischemic contracture), leading to a fixed flexed posture of the fingers. The mechanism of a clenched fist is overactivity of finger flexors, often with wrist flexors. This can result from muscle shortening (contractures), tendon shortening (injury/inflammation), or flexor muscle hyperactivity from diverse causes such as dystonia, spasticity, and neuromyotonia. The MP and IP joints can become ankylosed with prolonged clenched fist posture, resulting in a fixed flexion deformity. 

**Table 3:** Conditions Where Patients May Present With a Clenched Fist

Conditions
Spasticity
Stroke
Brain injuries
Cerebral palsy
Dystonia
Parkinson’s disease
Corticobasal syndrome
Lewy body dementia
Focal dystonia of other causes
Complex regional pain syndrome
Other nerve/muscle disorders
Focal neuromyotonia
Nerve injury/compression
Muscle injury/contracture
Psychiatric conditions
Functional neurological disorders
Schizophrenia

In Parkinson’s disease, patients have evidence of rigidity, bradykinesia, and/or a 4-6/second resting tremor. Table 4 highlights the findings that differentiate a claw hand due to ulnar neuropathy from a partial clenched fist [21]. 

**Table 4:** Differentiating a Claw Hand From a Partial Clenched Fist IP: interphalangeal; MP: metacarpophalangeal; EMG: electromyography; ADM: abductor digiti minimi; FDI: first dorsal interosseous.

Findings	Ulnar Neuropathy (Ring and Little Fingers)	Partial Clenched Fist (Any Two to Four Digits)
Distal IP joint	Flexed	Flexed
Proximal IP joint	Flexed	Flexed
MP joint	Hyperextended	Flexed
EMG denervation/reinnervation	ADM, FDI	No

Additionally, several pertinent features distinguish a finger drop due to a posterior interosseous neuropathy from a clenched fist (Table 5). 

**Table 5:** Differentiating Finger Drop From Clenched Fist IP: interphalangeal; MP: metacarpophalangeal; EMG: electromyography; EDC: extensor digitorum communis; EPL: extensor pollicis longus; EI: extensor indicis.

Finding	Posterior Interosseus Neuropathy	Clenched Fist
Distal IP joint	Extended	Flexed
Proximal IP joint	Extended	Flexed
MP joint	Flexed	Flexed
EMG denervation/reinnervation	EDC, EPL, EI	No

CFS is usually a diagnosis of exclusion and lacks positive radiographic, EDX, and laboratory findings [1]. Determining the etiology of clinical presentation with a clenched fist in a timely manner is imperative for determining the appropriate treatment. Treatment of patients with a clenched fist due to neurological conditions often involves unclenching of the hand under general anesthesia, dynamic splinting, stretching, botulinum toxin injections, and/or physical and occupational therapy of the hand [6,10]. In Cordivari and colleagues’ study of 14 patients with a “dystonic clenched fist” treated with botulinum toxin A, all had a significant decrease in pain, muscle spasm, and palmar infection (when present) [12]. Four patients with Parkinson’s disease and one patient with dystonia-CRPS achieved functional benefit. Patients with upper motor neuron disorders who develop a clenched fist may benefit from botulinum toxin injection but may need additional measures such as muscle-tendon lengthening, tenotomies, tendon transfers, muscle origin release, and joint capsule or ligament releases [11,22].

Mental health support may be indicated for patients with CFS with measures such as cognitive behavioral therapy, biofeedback, hypnosis, electroconvulsive therapy, or transcranial magnetic stimulation [6,16,23], although the prognosis is often poor due to patients’ lack of insight into the psychiatric nature of their disease [6]. Patients with CFS often do not comprehend the severity of their illness and are not willing to accept any additional psychiatric treatment. In a case report by Samir and colleagues, a patient with comorbid CFS and schizophrenia reportedly developed irreversible palmar contractures and thenar muscle atrophy, stressing the urgency of early intervention [16]. Instances of amputation of the affected hand have also been reported; the patient’s complaints prior to the amputation included pain, repeated infections, offensive smell, and cosmetic embarrassment [7,10]. Patients whose clenched fist resulted from trauma require prompt medical attention and potential surgical debridement or exploration of the deep structures [2].

In Weis and Boeckstyns’ series of 8 patients with CFS, all patients had sustained an injury or undergone surgery of the affected hand prior to the development of the clenched fist, 4 of whom had undergone an ulnar nerve decompression [7]. All cases were unilateral; 7 had involvement of the middle, ring, and little fingers; 4 had a psychiatric history; and 5 underwent amputations of more than one finger, all of whom developed painful contractures after the amputation. EMG and nerve conduction studies were performed in 4 patients who had symptoms of ulnar nerve compression.

Of the 17 patients in the current study, the etiology was known in 4 (spasticity and dystonia related to a lesion in the contralateral frontal lobe, Lewy body dementia, corticobasal degeneration, and tendinopathy). The clenched fist was idiopathic in 13 patients. Most of the patients in the latter category either had sustained a recent injury of the upper extremity with subsequent surgery or had an extensive psychiatric history, both of which are often inciting factors in developing a clenched fist. Most clenched fists were unilateral in our study, and the majority were partial clenched fists.

Physicians in several specialties, especially hand surgeons, refer patients with a clenched fist for neurodiagnostic assessment to our laboratory. The patients undergo neurological evaluations followed by EMG. EDX findings are analyzed in the context of the clinical picture. An abnormal study indicating neuromuscular disorders can be helpful in narrowing the differential diagnosis (confirming ventral horn cell, nerve root, peripheral nerve, and muscle disorders). If the EDX study is normal and there are signs of central nervous system disorders, the patient usually undergoes MR imaging, which may confirm or rule out conditions such as corticobasal syndrome and cerebrovascular disease. Parkinson's disease is a clinical diagnosis, but if a clenched fist is the only or main feature, additional workup like a DaT scan may be necessary. Thus, EDX coupled with a detailed neurological examination and appropriate imaging can diagnose the cause of a clenched fist in many cases. There may still be many patients in whom all studies are negative, in which case a functional neurological disorder becomes a presumptive diagnosis and referral for psychological evaluation is pursued.

In 1882, Stradling attributed “clenching of the hands” to “the result of mental emotion, of hysteria, or other nervous convulsion, of acute poisoning or of tetanus” [24]. Individuals may clench their fists as a subconscious physical manifestation of stress, aggression, or the need for self-control [24,25]. Psychological reasons for clenching fists include aggression and anger (“fight or flight” response), willpower and focus, stress and anxiety, and brain stimulation [23,24]. Unilateral hand clenching reportedly increases neuronal activity of the frontal lobe of the contralateral hemisphere [26]. Right hand clenching (left hemisphere activation; “approach” emotions such as happiness or anger) is associated with pre-encoding, while left hand clenching (right hemisphere activation; memory recall and withdrawal emotions) is linked to pre-recall [26].

The implications of our study are that physicians will become aware of the multiple causes of a clenched fist and develop an algorithm for evaluation and management of these patients. Prospective studies of larger cohorts of patients with a clenched fist are warranted to identify the cause and explore treatment options.

Strengths and limitations

The strength of the present study is that we describe a cohort of patients presenting with a clenched fist and highlight the need to be aware that causes other than somatic symptom disorder should also be considered and appropriate investigations pursued. Limitations of this study include its retrospective nature and lack of adequate follow-up. Most patients were evaluated on only one occasion at our Neurodiagnostic Center, and no data on the outcome of the disorder could be obtained. Therefore, we were unable to assess whether diagnostic timing changed treatment, clinical outcomes, or time to appropriate therapy. Another limitation of our study is that it comprised a highly selected cohort of 17 patients referred specifically to an EDX laboratory over 18.5 years. It was not a population-based sample of patients presenting with a clenched fist. Referral patterns may substantially influence the observed distribution of etiologies, and the findings in this study may not be representative of clenched fist presentations more generally. Additional limitations of the current study include the small sample size, single-center EDX referral population, exclusion of patients unable to provide a comprehensive history, and uncertain psychiatric diagnostic ascertainment in certain patients, which limit generalizability.

## Conclusions

A clenched fist is a clinical sign that may have varied etiologies, including psychiatric and neurological disorders, or may occur after localized trauma. The clenched fist may be partial or complete and is usually unilateral. In this study of a cohort of patients with a clenched fist, we sought to determine the underlying etiology. It is important to be aware that a clenched fist is not always psychosomatic in nature but may also be the result of a neurological non-factitious process. A prompt determination of the underlying etiology of the clenched fist may hasten appropriate treatment.
